# MYC sensitises cells to apoptosis by driving energetic demand

**DOI:** 10.1038/s41467-022-32368-z

**Published:** 2022-08-09

**Authors:** Joy Edwards-Hicks, Huizhong Su, Maurizio Mangolini, Kubra K. Yoneten, Jimi Wills, Giovanny Rodriguez-Blanco, Christine Young, Kevin Cho, Heather Barker, Morwenna Muir, Ania Naila Guerrieri, Xue-Feng Li, Rachel White, Piotr Manasterski, Elena Mandrou, Karen Wills, Jingyu Chen, Emily Abraham, Kianoosh Sateri, Bin-Zhi Qian, Peter Bankhead, Mark Arends, Noor Gammoh, Alex von Kriegsheim, Gary J. Patti, Andrew H. Sims, Juan Carlos Acosta, Valerie Brunton, Kamil R. Kranc, Maria Christophorou, Erika L. Pearce, Ingo Ringshausen, Andrew J. Finch

**Affiliations:** 1 Cancer Research UK Edinburgh Centre, Institute of Genetics and Cancer, University of Edinburgh, Crewe Road South, Edinburgh, EH4 2XR UK; 2 Department of Immunometabolism, Max Planck Institute of Immunobiology and Epigenetics, Stübeweg 51, D–79108 Freiburg, Germany; 3 Wellcome Trust/MRC Cambridge Stem Cell Institute & Department of Haematology, University of Cambridge, Cambridge, CB2 0AH UK; 4 Barts Cancer Institute, Queen Mary University of London, Charterhouse Square, London, EC1M 6BQ UK; 5 MRC Human Genetics Unit, Institute of Genetics and Molecular Medicine, University of Edinburgh, Crewe Road South, Edinburgh, EH4 2XR UK; 6 Department of Chemistry and Medicine, Washington University School of Medicine, St. Louis, MO 63110 USA; 7 MRC University of Edinburgh Centre for Reproductive Health, University of Edinburgh, Edinburgh, EH16 4TJ UK; 8 Centre for Genomic and Experimental Medicine, Institute of Genetics and Molecular Medicine, University of Edinburgh, Crewe Road South, Edinburgh, EH4 2XR UK; 9 MRC Center for Regenerative Medicine, University of Edinburgh, Edinburgh, EH8 9YL UK; 10 Present Address: Instituto de Biomedicina y Biotecnología de Cantabria, IBBTEC (CSIC, Universidad de Cantabria). C/ Albert Einstein 22, Santander, 39011 Spain; 11 Present Address: Department of Oncology, The Bloomberg-Kimmel Institute for Cancer Immunotherapy, Johns Hopkins University, Baltimore, MD USA

**Keywords:** Cancer metabolism, Apoptosis, Oncogenes

## Abstract

The *MYC* oncogene is a potent driver of growth and proliferation but also sensitises cells to apoptosis, which limits its oncogenic potential. MYC induces several biosynthetic programmes and primary cells overexpressing *MYC* are highly sensitive to glutamine withdrawal suggesting that MYC-induced sensitisation to apoptosis may be due to imbalance of metabolic/energetic supply and demand. Here we show that MYC elevates global transcription and translation, even in the absence of glutamine, revealing metabolic demand without corresponding supply. Glutamine withdrawal from MRC-5 fibroblasts depletes key tricarboxylic acid (TCA) cycle metabolites and, in combination with MYC activation, leads to AMP accumulation and nucleotide catabolism indicative of energetic stress. Further analyses reveal that glutamine supports viability through TCA cycle energetics rather than asparagine biosynthesis and that TCA cycle inhibition confers tumour suppression on MYC-driven lymphoma in vivo. In summary, glutamine supports the viability of MYC-overexpressing cells through an energetic rather than a biosynthetic mechanism.

## Abstract (web summary)

MYC activation can sensitise cells to apoptosis upon glutamine withdrawal. Here the authors show that MYC activation enhances global transcription and translation that creates a metabolic demand, while glutamine limitation causes a metabolic demand and supply imbalance through loss of TCA energetics and thus, sensitises cells to apoptosis.

## Introduction

The *MYC* oncogene is a potent driver of growth and proliferation, and these oncogenic attributes drive its amplification or overexpression in many human cancers^1^. Deregulation of *MYC* also drives activation of tumour suppressive pathways, most notably sensitisation to apoptosis^2^ and activation of the p53 pathway resulting in restriction of cell cycle^3,4^. Deregulation of *MYC* sensitises cells to multiple apoptotic triggers in vitro, including withdrawal of serum^2^, treatment of cells with death receptor ligands such as TNF^5^ or TRAIL^6^ and withdrawal of glutamine^7^. MYC-induced sensitisation to apoptosis is abrogated in most systems upon expression of antiapoptotic members of the BCL-2 family, such as BCL-2 or BCL-xL^8^. Similarly, many systems show a requirement for one or both of the proapoptotic BCL-2 family members BAX or BAK1 for the apoptosis induced by MYC^9–11^, although the molecular mechanism is not thought to involve direct transcriptional activation of these genes.

Two conceptual models of MYC-induced transcription have been proposed. The longest-standing model is that MYC activates a specific set of target genes, estimated to represent over 10% of the mammalian genome under conditions of MYC overexpression^12^ and that these target genes mediate both its tumorigenic and tumour suppressive functions. More recently, two studies provided comprehensive analyses of MYC-induced gene targets and promoter occupancy in various contexts^13,14^, allowing unparalleled insight into the regulation of gene expression afforded by MYC. A contrasting model holds that MYC activation acts to amplify the existing transcriptional programme of the cell^15,16^ and such a model would be hard to reconcile with one in which the apoptotic programme of MYC was based upon specific gene targets. Regardless of which model is correct, no convincing MYC target genes have thus far been identified that carry out its proapoptotic function.

In addition to activation of RNA polymerase II-directed transcription, MYC activates transcription directed by both RNA polymerases I and III^17–20^, which impacts upon the total cellular content of RNA as well as transcriptional and translational load. This potent deregulation of global transcription requires the provision of abundant nucleotide triphosphates (NTPs). This, in turn, creates considerable demand for the energy and metabolic precursors required to feed the pentose phosphate pathway, pathways of purine and pyrimidine nucleotide biosynthesis and synthesis and function of the translational machinery. Indeed, several groups have reported MYC-induced activation of genes in the pentose phosphate and nucleotide biosynthesis pathways in vitro^21–26^ and in vivo in *D. melanogaster*^27^ and in mouse tumours^13,14,28^.

Using untargeted stable isotope tracing of glucose and glutamine^29^, we identified nucleotide catabolism as an unexpected consequence of MYC activation. Nucleotide catabolism is known to occur in multiple tissues under conditions of hypoxia, particularly in muscle when metabolic demand exceeds supply^30–33^ and this led us to hypothesise that MYC may elicit high metabolic demand and that apoptosis may occur when metabolic supply does not meet that demand. Here, we report that MYC drives a striking increase in global transcription and translation and accordingly diverts glycolytic carbon from 3-phosphoglyceraldehyde to ribose 5-phosphate via reversal of the non-oxidative pentose phosphate pathway. This high demand for nucleotides provides a platform for the glutamine-dependence of cells harbouring deregulated MYC^7,34^ and we therefore investigated this dependence in the context of metabolic supply and demand. Withdrawal of glutamine in MYC-overexpressing primary human MRC-5 fibroblasts led to a near-complete loss of key TCA cycle metabolites and a striking increase in AMP, ADP and nucleoside/nucleobase levels and activation of AMP kinase, indicating energetic stress due to lack of TCA cycle activity. Withdrawal of serum, which also caused depletion of TCA cycle metabolites, triggered apoptosis in these cells, as did pharmacological inhibition of the TCA cycle enzyme oxoglutarate dehydrogenase (OGDH). Indeed, the latter also elicited tumour suppression in MYC-driven lymphoma in vivo. Perturbations of mitochondrial energetic supply and cellular metabolic demand also revealed an inverse relationship between energetic status and apoptosis. Finally, we showed that asparagine supplementation rescued these cells from apoptosis but so did the knockdown of asparagine synthetase (ASNS), indicating that exogenous asparagine protects from apoptosis through product inhibition of ASNS activity rather than through enabling protein synthesis, as previously suggested^34^. Taken together, our results demonstrate that MYC-induced sensitisation to apoptosis occurs through an imbalance between metabolic demand and mitochondrial energy supply.

## Results

### MYC drives continued metabolic demand after withdrawal of glutamine

**Fig. 1:**
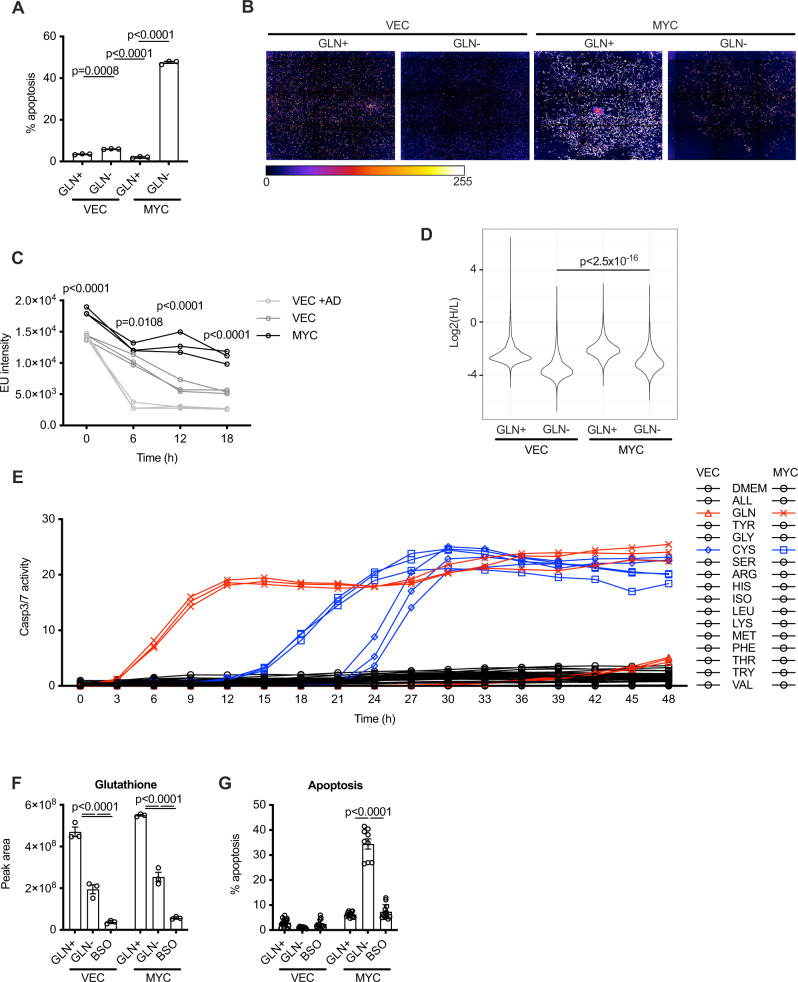
MYC drives continued metabolic demand after withdrawal of glutamine. **A** MRC-5 cells expressing MYC-ER^T2^ (MYC) or vector control (VEC) were stimulated for 24 h with 200 nM 4-OHT, and glutamine was then withdrawn for 16 h (GLN−). Apoptosis was assessed by annexin V/PI staining. An ordinary one-way ANOVA was used to determine statistical significance (*n* = 3 biological replicates, representative of three independent experiments). **B** Cells were treated with 4-OHT for 24 h, and glutamine was then withdrawn for 12 h. A 30 min pulse with 5-ethynyl uridine (EU) revealed nascent transcripts. EU incorporation was revealed with a fluorescent azide (with DAPI counterstained). **C** High-content imaging of EU incorporation at the stated times in cells treated as above or with 500 μM actinomycin D. A two-way ANOVA was used to determine statistical significance (*n* = 3 biological replicates). **D** Measurement of global translation through ^13^C_6_,^15^N_2_-lysine incorporation and shotgun proteomics. **E** Cells were switched to DMEM lacking the indicated amino acids and apoptosis was quantified by Incucyte caspase 3/7 analysis. A two-way ANOVA was used to determine statistical significance (*n* = 3 biological replicates). **F** LC-MS quantitation of glutathione in cells following 16 h glutamine withdrawal or BSO treatment. A two-way ANOVA was used to determine statistical significance (*n* = 3 biological replicates). **G** AnnexinV/PI staining in cells treated as in (**F**). A two-way ANOVA was used to determine statistical significance (*n* = 3 biological replicates). For all statistical tests, *P* ≤ 0.05 was considered significant, and error bars show the standard error of the mean.

The study of transcriptional targets of MYC has revealed a great deal about the biology of this oncogene. However, transcriptional approaches cannot interrogate homeostatic responses to MYC activation that, by definition, are secondary to its primary transcriptional function. We hypothesised that the apoptotic function of MYC arises due to metabolic imbalance and we were particularly interested in homeostatic responses to global transcriptional deregulation for reasons described above. We chose primary human fibroblasts (MRC-5) for these experiments because they are genetically normal, and all tumour suppressive and homeostatic pathways are therefore intact. MRC-5 fibroblasts expressing MYC-ER^T2^ (from the retroviral vector pM6P) in the presence of 4-hydroxytamoxifen (4-OHT) are highly sensitive to glutamine withdrawal and undergo apoptosis, as measured by annexin V/propidium iodide staining (Fig. 1A and Supplementary Fig. 1a). We confirmed that this cell death was indeed apoptotic by abrogating it through overexpression of BCL-xL (Supplementary Fig. 1b). We also verified that Myc-driven cancer cells (from an Eμ-Myc mouse lymphoma) were sensitive to glutamine withdrawal (Supplementary Fig. 1c), as previously reported for MYC-driven glioma cells^35^. Re-addition of glutamine rescued Myc-overexpressing cells from the apoptotic phenotype and enabled their proliferation, consistent with a homeostatic mechanism based on metabolic supply and demand rather than triggering of an irreversible commitment to apoptosis and cell cycle arrest (Supplementary Fig. 1d).

Glutamine, glutamine-derived aspartate and energy are all required for NTP biosynthesis and we therefore reasoned that withdrawal of glutamine should be incompatible with continued usage of nucleotides for transcription. Cells transduced with pM6P vector responded to glutamine withdrawal by rapidly reducing their global transcription (Fig. 1B, C) in a homeostatic response to the withdrawal of a key nutrient. In cells transduced with pM6P-MYC-ER^T2^, MYC activation strongly enhanced global transcription in cells under normal growth conditions (Fig. 1B). This demonstrates that deregulated MYC exerts a remarkable transcriptional drive over and above that of 10% foetal calf serum (itself a potent driver of transcriptional activation). However, with deregulated MYC, elevated transcription was maintained even in the absence of glutamine such that EU incorporation almost matched that in vector control cells cultured under full growth conditions (Fig. 1B, C).

Protein translation exerts demand for glutamine as a biosynthetic precursor and energy source to fuel the GTP-dependent reactions of polypeptide chain elongation. Promotion of global translation by MYC has previously been reported^36^ and we wished to assess how glutamine withdrawal coupled with MYC deregulation would impact upon this process. We used ^13^C_6_,^15^N_2_-lysine labelling and shotgun proteomic analysis by LC-MS to measure rate of global translation. In parallel with our observed increase in global transcription, we found that MYC activation led to elevated translation throughout the proteome (Fig. 1D). Withdrawal of glutamine reduced global translation in both conditions, but there was a highly significant increase in translation driven by MYC compared to that seen in cells transduced with the pM6P vector in the absence of glutamine (*P* value = 2.2 × 10^−16^). Thus MYC can drive high and continued demand for glutamine and glutamine-derived metabolites for biosynthetic and energetic purposes even when glutamine is withdrawn.

### Glutamine withdrawal does not sensitise MYC-expressing cells to apoptosis through inhibition of translation or glutathione synthesis

We investigated the amino acid specificity of the apoptotic response through withdrawal of individual amino acids from the medium (Fig. 1E). MYC activation rapidly sensitised the cells to the withdrawal of glutamine whilst vector cells showed no signs of apoptosis. A more delayed induction of apoptosis was also observed in cells transduced with either pM6P or pM6P-MYC-ER^T2^ that were deprived of cystine, although this was slower than the response to glutamine deprivation and only slightly accelerated by MYC activation. Withdrawal of the other amino acids did not lead to apoptosis in either cell type as previously reported^7^ nor did glutamine withdrawal from cells transduced with the pM6P vector, most likely because of homeostatic attenuation of biosynthetic processes in response to the withdrawal of an essential nutrient. We therefore conclude that glutamine withdrawal does not trigger apoptosis in MYC-expressing cells through impairment of translation. Important cytoprotective metabolic fates of glutamine and cystine are into the biosynthesis of glutathione, which acts to limit ROS-mediated damage and apoptosis. Treatment of cells with 20 μM l-buthionine sulfoximine (BSO), a potent inhibitor of glutathione synthesis, led to a more striking drop in glutathione levels than glutamine withdrawal (Fig. 1F). However, BSO treatment did not act as a trigger for apoptosis in MYC-expressing cells (Fig. 1G), indicating that the cytoprotective function of glutamine was not through the production of glutathione to protect cells from ROS, consistent with a previous report^7^.

### MYC drives nucleotide biosynthesis and catabolism

Having established high MYC-driven metabolic demand, we wished to interrogate metabolite supply in more detail and we chose untargeted stable isotope approaches to examine the usage of two key nutrients, glucose and glutamine, upon MYC activation. Cells transduced with pM6P or pM6P-MYC-ER^T2^ were labelled for 6 hours with stable isotopes after activation of MYC-ER^T2^ with 4-OHT. Extracts from the cells were subjected to LC-MS and then X13CMS software^29^ was used to identify statistically significant changes in label incorporation into metabolites in an untargeted fashion. Once metabolites were identified in X13CMS, we performed targeted analyses to examine other metabolites from the corresponding metabolic pathways (Supplementary Fig. 2 and Supplementary Tables 1 and 2). Glucose-derived carbon was increased by MYC activation in several metabolites of core carbon metabolism, supporting a general biosynthetic role for MYC. Far more striking, however, were the changes observed using ^15^N-amide-glutamine, for which MYC activation led to increased incorporation into NTPs, as expected, but also into nucleosides (e.g., cytidine: 11-fold), nucleobases (e.g., uracil: 30-fold) and nucleotide-containing lipid precursors (Supplementary Fig. 2 and Supplementary Tables 1 and 2). Nucleotides are comprised of a nucleobase, a ribose sugar and one or more phosphate groups, whereas nucleosides (a nucleobase and the ribose sugar) and nucleobases (no phosphates or ribose) are produced by catabolism of nucleotides or extracellular uptake. The presence of label in nucleosides and bases therefore indicates both nucleotide synthesis and catabolism during the period of labelling. ^13^C_6_-glucose was also incorporated into nucleotides as well as other nucleotide derivatives, albeit to a lesser extent. We found no significant changes with ^13^C_5_-glutamine in these experiments, largely because abundant labelling of the TCA cycle was observed regardless of MYC activation (see below). These data indicate that the main metabolic change elicited by MYC activation is a reprogramming of nucleotide metabolism. In particular, they reveal an unexpected MYC-dependent induction of nucleotide catabolism in addition to induction of nucleotide biosynthesis.

### MYC drives nucleotide production through salvage of bases in the absence of glutamine

**Fig. 2:**
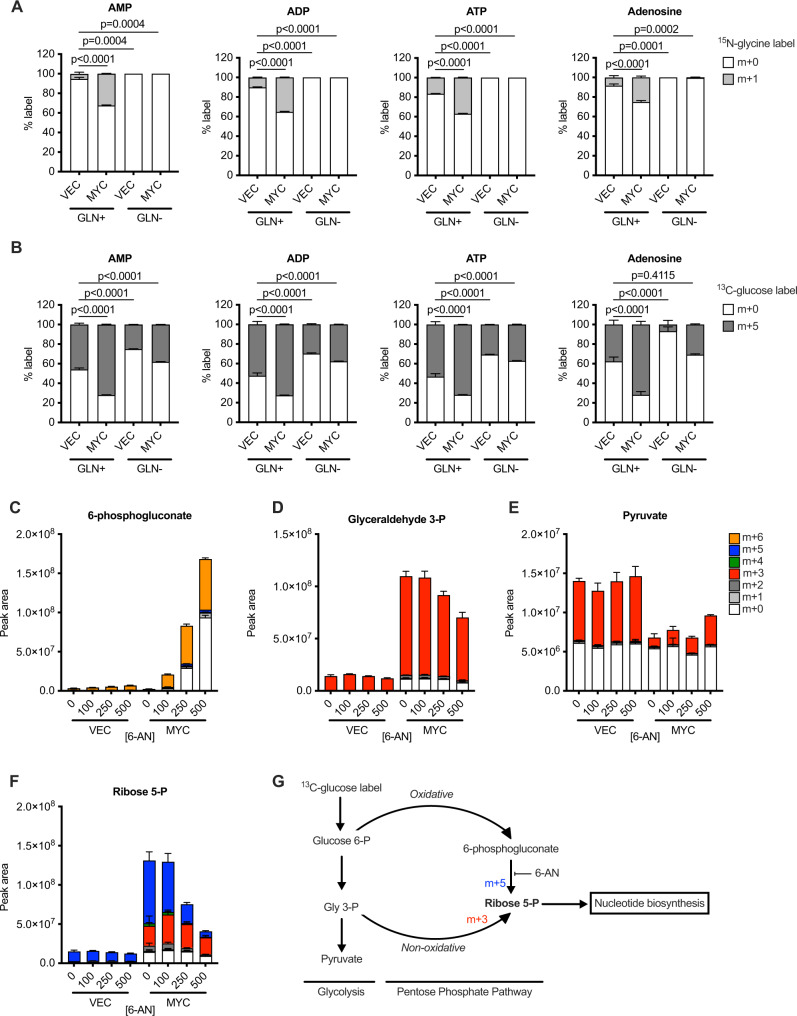
MYC drives nucleotide production through the salvage pathway in the absence of glutamine and causes reversal of the non-oxidative pentose phosphate pathway. **A**, **B** MRC-5 cells expressing MYC-ER^T2^ (MYC) or vector control (VEC) were labelled with (**A**) ^15^N-glycine for 12 h or (**B**) ^13^C_6_-glucose for 6 h and the indicated metabolites and isotopologues were measured by LC-MS. An ordinary one-way ANOVA was used to determine statistical significance (*n* = 6 biological replicates). **C**–**F** Cells were treated with 6-AN at the doses shown (in μM), and a 5 min pulse with ^13^C_6_-glucose was used to visualise flux into (**C**) 6-phosphogluconate, (**D**) glyceraldehyde 3-phosphate, (**E**) pyruvate, and (**F**) ribose 5-phosphate. **G** Scheme showing how MYC reroutes glycolytic carbon to support nucleotide biosynthesis. For all statistical tests *P* ≤ 0.05 was considered significant, and error bars show the standard error of the mean.

The amplification of global transcription and elevation of nucleotide biosynthesis by MYC led us to investigate nucleotide biosynthesis pathways in more detail. The de novo synthesis of nucleotides occurs through series of enzymatic reactions that utilise ATP, tetrahydrofolate derivatives, glutamine, glycine, aspartate and phosphoribosyl pyrophosphate. Targeted analyses revealed that MYC activation led to increased incorporation of the amide nitrogen from glutamine into nucleotide monophosphates (NMPs) (Supplementary Fig. 2 and Supplementary Tables 1 and 2), suggesting that removal of glutamine might inhibit nucleotide biosynthesis and thereby simultaneously remove demand for energy and biosynthetic precursors. Indeed, ^15^N-glycine labelling experiments revealed that withdrawal of glutamine inhibited de novo production of adenine nucleotides and adenosine (Fig. 2A). However, labelling of cells with ^13^C_6_-glucose revealed ongoing production of adenine nucleotides containing labelled ribose (m + 5 isotopologue) upon glutamine withdrawal, and this was significantly higher in cells transduced with pM6P-MYC-ER^T2^ (Fig. 2B). The nucleotide salvage pathway is essentially a reversal of the nucleotide/nucleoside catabolism pathway and is employed by cells to conserve or recover nitrogen in the form of nucleobases. Our identification of nucleotides containing labelled ribose indicates that in the absence of glutamine, MYC can still produce nucleotides through the pentose phosphate pathway coupled with salvage of nucleobases and subsequent activity of the nucleotide kinases.

The salvage route for NTP biosynthesis still maintains the energetic demand of glucose phosphorylation to feed the pentose phosphate pathway and the kinases required to phosphorylate NMPs to NTPs. We used a very short (5 min) pulse of ^13^C_6_-glucose along with 6-aminonicotinamide (6-AN: an inhibitor of 6-phosphogluconate dehydrogenase) to visualise metabolic flux through the pentose phosphate pathway. MYC activation led to a striking increase in 6-phosphogluconate levels and label incorporation (m + 6) from ^13^C_6_-glucose in the presence of 6-AN, revealing high MYC-driven flux through the pathway (Fig. 2C). We observed a strong MYC-induced increase in G3P levels, and there was a high proportion of G3P labelled with the m + 3 isotopologue (Fig. 2D). In contrast, the amount of (m + 3) pyruvate that we observed with this short pulse was much lower upon MYC activation (Fig. 2E). A dramatic increase in ribose 5-phosphate levels was also observed upon MYC activation, but not in control cells transduced with pM6P vector, and this was reduced by 6-AN (Fig. 2F). The isotopologue distribution of ribose 5-phosphate showed that the majority of MYC-driven synthesis was via the oxidative arm (m + 5), however, there was a proportion that was labelled m + 3. Whilst the oxidative (m + 5) contribution was sensitive to 6-AN, the m + 3 contribution was not, indicating that it arose through the reversal of the non-oxidative arm of the pathway from glyceraldehyde 3-phosphate (G3P). Taken together, these data indicate that the demand for ribose 5-phosphate driven by MYC is so strong that it reroutes glycolytic carbon through a reversed non-oxidative PPP (Fig. 2G).

### Nucleotide catabolism indicates energetic imbalance driven by MYC

**Fig. 3:**
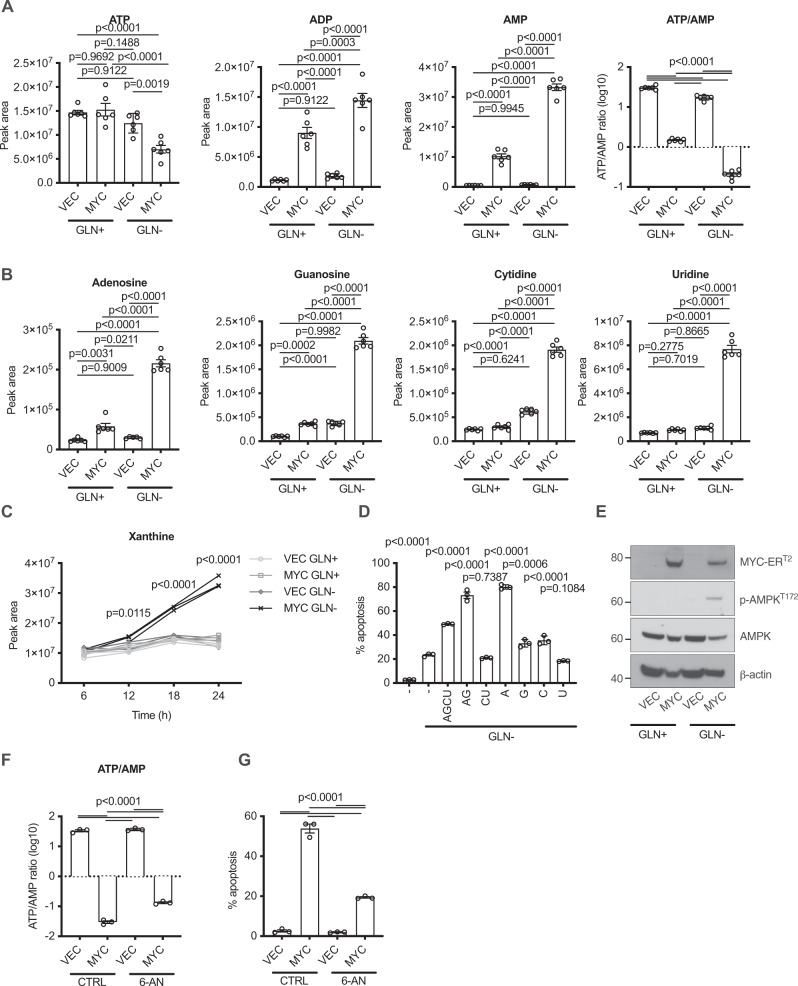
Energetic crisis and nucleotide catabolism are driven by MYC in the absence of glutamine. LC-MS was used to quantify levels of metabolites in MRC-5 cells treated for 24 h with 200 nM 4-OHT and then glutamine withdrawal for 16 h. **A**, **B** Bar charts show peak intensities of nucleotides, nucleosides, and the ratio of ATP/AMP peak intensities. An ordinary one-way ANOVA was used to determine statistical significance (*n* = 6 biological replicates, representative of three independent experiments). **C** Time course of xanthine levels (glutamine withdrawn at time 0). A two-way ANOVA was used to determine statistical significance (*n* = 3 biological replicates, representative of three independent experiments). **D** AnnexinV/PI assay of MYC cells with the addition of the indicated nucleosides (1 μM total). Significance is relative to Ctrl—Gln, calculated using an ordinary one-way ANOVA (*n* = 3). **E** Western blot for MYC, phospho-AMPK, total AMPK and β-actin. **F** Ratio of ATP/AMP peak intensities of cells containing MYC-ER^T2^ (MYC) or vector control (VEC) deprived of glutamine upon inhibition of the PPP with 250 μM 6-AN. An ordinary one-way ANOVA was used to determine statistical significance (*n* = 3 biological replicates, representative of three independent experiments). **G** AnnexinV/PI assay on cells as in (**F**). For all statistical tests *P* ≤ 0.05 was considered significant, and error bars show the standard error of the mean.

Whilst nucleotide metabolism is a logical biosynthetic programme for MYC to engage, the concurrent nucleotide catabolism seems paradoxical. Physiological states in which nucleotide catabolism is observed are ischaemia^31^ and in the exercise of muscle, particularly under conditions of ischaemia^30,33^. These are states in which energetic supply cannot meet demand, and we therefore examined whether MYC might promote nucleotide catabolism through high energy consumption, particularly when energetic supply is limiting (e.g. upon glutamine withdrawal). We found that cellular ATP levels were slightly lower upon glutamine withdrawal, and these were further reduced by MYC activation (Fig. 3A), although reasonable levels of ATP were maintained. However, whilst AMP and ADP levels were not altered by glutamine withdrawal in control cells transduced with pM6P vector, in cells transduced with pM6P-MYC-ER^T2^, the combination of MYC activation and glutamine withdrawal led to a striking increase in these nucleotides (Fig. 3A). Furthermore, the combination of MYC activation and glutamine withdrawal led to the accumulation of the four nucleosides adenosine, guanosine, cytidine and uridine (Fig. 3B). Further breakdown products of purine nucleosides include hypoxanthine, which is strongly elevated during ischaemia in vivo^31^, and xanthine. Xanthine is produced from hypoxanthine through the action of xanthine oxidase (XDH) in a reaction that also releases hydrogen peroxide. The rapid clearance of hydrogen peroxide by peroxidases and catalases renders this reaction irreversible under physiological conditions, and this reaction therefore leads to the loss of purines from the cellular metabolic pool. We observed xanthine accumulation only under conditions of MYC activation and glutamine withdrawal (Fig. 3C). This raised the possibility that nucleobase availability could be limiting under apoptotic conditions due to irreversible catabolism and so we added exogenous nucleosides to the cells to assess whether this would protect from apoptosis. The addition of all four nucleosides together did not rescue the cells from apoptosis but rather enhanced it (Fig. 3D). Addition of separate nucleosides indicated that adenosine was primarily responsible for the enhancement of apoptosis, and no reduction in apoptosis was seen with any of the nucleosides. These data, combined with the reduction in energetic status that we observed, indicate that the availability of nucleobases is not limiting under conditions of MYC activation and glutamine withdrawal. The changes in nucleotide/nucleoside/nucleobase levels observed upon MYC overexpression and glutamine withdrawal also led to activation of AMP kinase (Fig. 3E), indicating that the reduction in cellular energy charge represented energetic stress sufficient to trigger a homeostatic cellular response.

As described above, MYC drives elevated flux through the PPP to support the production of ribose 5-phosphate for nucleotides. We hypothesised that reduction of PPP activity may reduce metabolic demand and thereby reduce the extent of apoptosis. Inhibition of the PPP with 6-AN reversed the MYC-driven energetic defect (Fig. 3F), and this was accompanied by a strong reduction in apoptosis (Fig. 3G). In order to verify that the reduced apoptosis was caused by the reduction in energetics, rather than vice versa, we examined energy charge shortly after the withdrawal of glutamine prior to the onset of apoptosis. Activation of MYC and withdrawal of glutamine for one hour caused a drop in energy balance in a similar manner to that observed at later time points (Supplementary Fig. 3a). When cells with activated MYC were treated with 6-AN prior to glutamine withdrawal for one hour, improved cellular energy balance was observed (Supplementary Fig. 3b), indicating that the changes in energy preceded apoptosis rather than being caused by its onset, which occurs after 3 h of glutamine withdrawal (Fig. 1E). Thus, through driving metabolic demand, MYC sensitises cells to the withdrawal of mitochondrial energetic support.

### MYC drives nucleotide remodelling in cancer cells

**Fig. 4:**
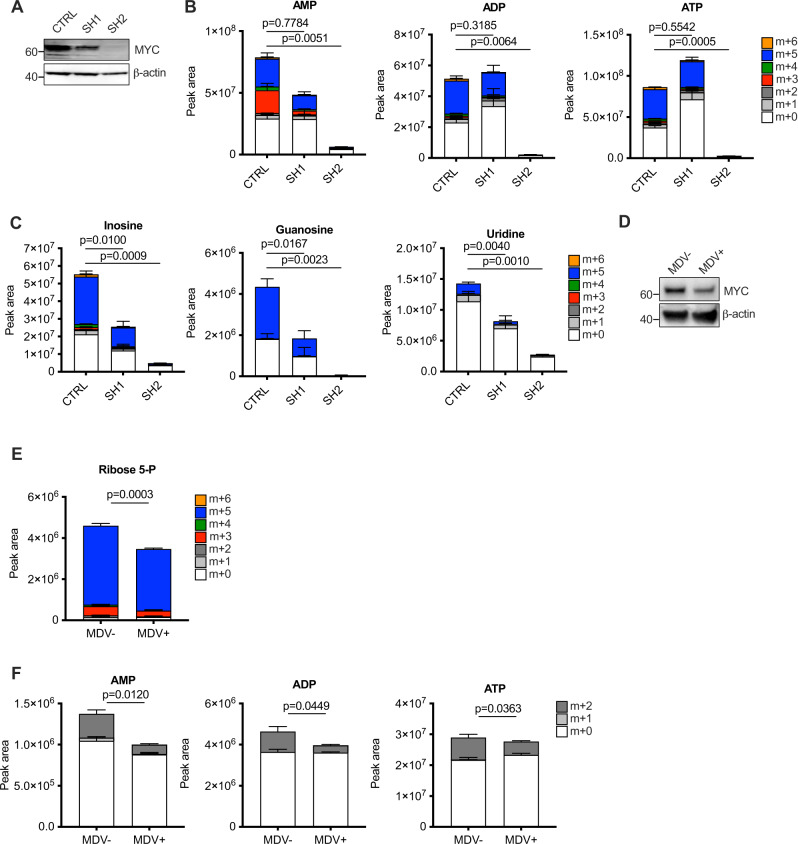
Induction of nucleotide biosynthesis, catabolism and PPP remodelling is dependent upon MYC in MYC-driven cancer cells. LC-MS was used to quantify isotopologues of metabolites following ^13^C_6_-glucose pulse in RAJI Burkitt’s lymphoma (**A**–**C**) and Myc-caP mouse prostate cancer cells (**D**–**F**). **A** Western blot showing shRNA-mediated knockdown of MYC in Raji cells. **B**, **C** Incorporation of ^13^C_6_-glucose with a 6 h pulse into (**B**) adenine nucleotides and (**C**) nucleosides. An ordinary one-way ANOVA was used to determine statistical significance for the m + 5 isotopologue (*n* = 3 biological replicates). **D** Western blot showing reduction of MYC levels upon 96 h enzalutamide (MDV) treatment in Myc-caP cells. **E**, **F** Incorporation of (**E**) Incorporation of ^13^C_6_-glucose into ribose 5-phosphate with a 5 min pulse and (**F**) ^15^N-glutamine into adenine nucleotides with a 6 h pulse in Myc-caP cells treated for 48 h with enzalutamide (MDV). An ordinary one-way ANOVA was used to determine statistical significance for the m + 5 or m + 2 isotopologue for (**E**) and (**F**), respectively (*n* = 3 biological replicates). For all statistical tests, *P* ≤ 0.05 was considered significant and error bars show the standard error of the mean.

As described above, activation of MYC drove extensive remodelling of nucleotide metabolism in primary human fibroblasts, in particular, reversal of the non-oxidative arm of the pentose phosphate pathway and flux into nucleosides indicating catabolism. In order to assess whether MYC also drives these changes in cancer cells, we examined these processes in two cell systems where MYC levels were lowered in MYC-driven cancer cell lines. shRNAs to MYC were used in RAJI cells, a Burkitt’s lymphoma cell line in which MYC deregulation occurs due to a translocation of the *MYC* gene to the immunoglobulin heavy-chain locus. One shRNA (sh2) essentially abrogated *MYC* expression whilst a second (sh1) gave partial knockdown (Fig. 4A). ^13^C_6_-glucose labelling and analysis of AMP revealed equal contribution to the m + 5 (oxidative PPP) and m + 3 (reversed non-oxidative PPP) isotopologues in the parental line transfected with non-silencing shRNA control. Knockdown of MYC led to a reduction in the contribution of both pathways to AMP synthesis, with the sh2 shRNA leading to striking depletion of AMP, ADP and ATP (Fig. 4B). Similarly, the contribution of glucose-derived carbon to the nucleosides inosine, guanosine and uridine was strongly suppressed upon MYC knockdown, and sh2 again led to striking depletion (Fig. 4C). Importantly, in all of the products of nucleotide catabolism, the reduction in amount and contribution of label matched the extent of knockdown of MYC. The second system that we employed is the Myc-CaP mouse cancer cell line derived from mouse prostate tumours driven by MYC under the control of an androgen-responsive element^37^. Treatment of the cells with the androgen receptor antagonist enzalutamide (MDV) led to a reduction in expression of MYC (Fig. 4D), and a short (5 min) ^13^C_6_-glucose pulse revealed a corresponding decrease in the m + 3 and m + 5 isotopologues of ribose 5-phosphate (Fig. 4E). Similarly, a 6-h ^15^N-amide glutamine pulse revealed reduced nucleotide biosynthesis as measured by label incorporation (m + 2) into the adenine nucleotides (Fig. 4F). Thus the promotion of ribose 5-phosphate production through both forward oxidative and reversed non-oxidative pathways and the promotion of nucleotide biosynthesis and catabolism are driven by MYC in cancer cells.

### MYC sensitises cells to apoptosis triggered by the limitation of the TCA cycle

**Fig. 5:**
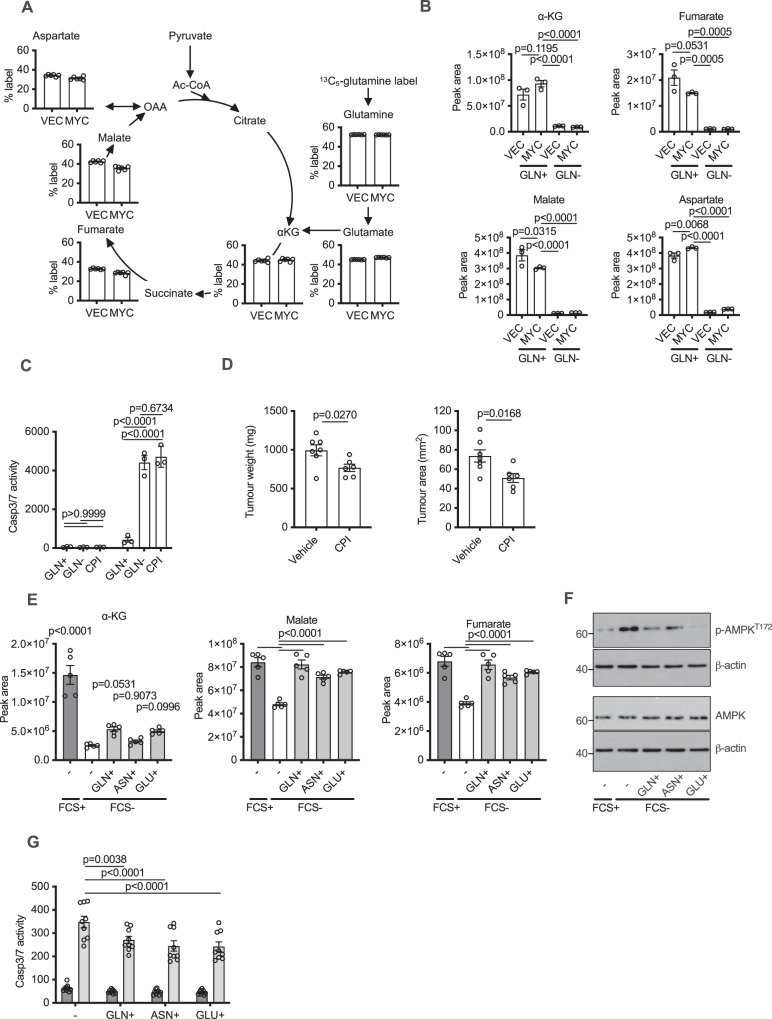
Loss of TCA cycle triggers MYC-induced apoptosis and reduces tumourigenesis in vivo. **A** Percent labelling of the oxidative TCA cycle in MRC-5 cells measured by LC-MS following a 6 h ^13^C_5_-glutamine pulse. The predominant oxidative TCA cycle isotopologue (m + 5 or m + 4) is shown for each TCA intermediate. **B** LC-MS measurement of TCA cycle intermediates. An ordinary one-way ANOVA was used to determine statistical significance (*n* = 3 biological replicates, representative of three independent experiments). **C** Incucyte caspase 3/7 apoptosis assay 24 h after treatments shown. CPI = CPI-613. **D** Weight and tumour area are shown in endpoint tumours from mice injected subcutaneously with 1 × 10^6^ Raji lymphoma cells and treated with vehicle or CPI-613 (20 mg/kg). Statistical analysis was carried out on 6–7 biological replicates using an unpaired two-tailed *t* test. **E** Peak areas of indicated metabolites 16 h after FCS withdrawal and with supplementation of indicated amino acids (10 mM). An ordinary one-way ANOVA was used to determine statistical significance (*n* = 5 biological replicates). **F** Western blots for phospho-AMPK, total AMPK and β-actin from samples as in (**e**) but at 16 h. **G** Incucyte caspase 3/7 apoptosis assay 24 h after treatments shown. An ordinary one-way ANOVA was used to determine statistical significance (*n* = 9 biological replicates). For all statistical tests, *P* ≤ 0.05 was considered significant, and error bars show the standard error of the mean.

Because of the lowered energy status and increased nucleotide catabolism, we investigated the integrity of the TCA cycle, the main driver of mitochondrial ATP generation. Through incubation of cells in ^13^C_5_-glutamine and measurement of derived metabolites, we observed abundant flux of glutamine-derived carbon into the TCA cycle (Fig. 5A). However, we did not observe significant reductive carboxylation driven by MYC, as assessed by the minimal presence of the m + 5 isotopologue of citrate following ^13^C_5_-glutamine labelling (Supplementary Fig. 4). Upon withdrawal of glutamine, we observed almost complete loss of the TCA cycle mediators α-ketoglutarate, fumarate and malate (Fig. 5B), indicating loss of TCA cycle and therefore a lack of generation of NADH to drive mitochondrial ATP production. Aspartate, a key precursor for pyrimidine synthesis and asparagine production, was also strongly depleted. In order to assess whether loss of TCA cycle activity was responsible for triggering MYC-induced apoptosis, we made use of the lipoate analogue drug CPI-613 to inhibit oxoglutarate dehydrogenase (OGDH)^38^ in place of glutamine withdrawal. CPI-613 can inhibit PDH and OGDH^38,39^, although its mechanism of action is not fully understood, and in some systems it has been reported to inhibit cell proliferation without inhibiting OGDH^38,40^. Treatment of MRC-5 fibroblasts with CPI-613 led to equivalent induction of apoptosis to glutamine withdrawal, and with similar kinetics, but only in those cells overexpressing activated MYC-ER^T2^ (Fig. 5C and Supplementary Fig. 5a). Furthermore, the addition of exogenous dimethylsuccinate to the medium significantly rescued the apoptosis triggered by CPI-613 (Supplementary Fig. 5b), indicating inhibition of OGDH in these cells by the drug. Targeting glutamine metabolism in cancers in vivo through pharmacological inhibition of glutaminase has yielded relatively modest results (reviewed in ref. 41), mainly because many enzymes besides glutaminase can convert glutamine into glutamate^7^. However, OGDH is the only mitochondrial enzyme that can convert α-ketoglutarate into succinyl-coA in the TCA cycle and may therefore represent a more promising therapeutic target for MYC-driven tumours. We tested whether CPI-613 could elicit tumour suppression in vivo in a MYC-driven B-cell lymphoma model initiated by subcutaneous injection of RAJI cells into mice. At experimental endpoint, the tumours from mice that had been treated with CPI-613 weighed significantly less than their untreated counterparts (Fig. 5D). Furthermore, the proportion of tissue that was comprised of tumour cells, as opposed to stroma or other surrounding tissue, was also significantly reduced (Fig. 5D). Our results suggest that directly targeting the TCA cycle, rather than glutamine catabolism, may be a promising therapeutic approach for MYC-driven tumours.

Serum withdrawal can also trigger MYC-induced apoptosis^2^, and so we examined whether loss of TCA cycle activity was also observed upon this apoptotic stimulus. LC-MS analysis of metabolites from MYC-overexpressing cells showed a striking drop in α-ketoglutarate levels due to FCS (foetal calf serum) withdrawal and also a reduction in levels of fumarate and malate (Fig. 5E). FCS withdrawal from these cells also led to phosphorylation of AMPK (Fig. 5F), indicating induction of energy stress as observed with glutamine withdrawal. The addition of high concentrations (10 mM) of glutamine, asparagine or glutamate elevated the levels of TCA metabolites in the FCS-deficient condition (Fig. 5E), reduced AMPK phosphorylation (Fig. 5F) and reduced apoptosis (Fig. 5G). Thus TCA cycle restriction and consequent energetic depletion is, at least in part, a mechanism through which FCS withdrawal triggers MYC-induced apoptosis.

### Glutamine supports viability through anaplerosis but not biosynthesis of asparagine

**Fig. 6:**
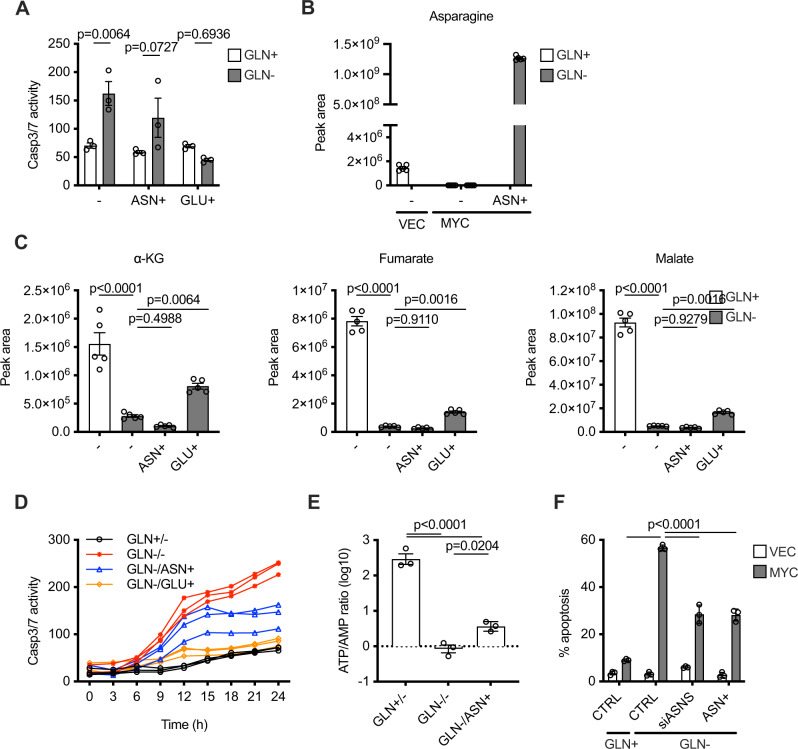
Glutamine withdrawal-induced apoptosis does not occur due to loss of asparagine. **A** Incucyte Caspase 3/7 apoptosis assay 24 h after treatments shown in 4-OHT-treated BJ fibroblasts expressing MYC-ER^T2^. Supplemented amino acids are at 4 mM. An ordinary one-way ANOVA was used to determine statistical significance (*n* = 3 biological replicates). **B** Peak area of asparagine 6 h after glutamine withdrawal and asparagine supplementation in cells as in (**A**). **C** Peak areas of indicated metabolites 6 h after glutamine withdrawal and amino acid supplementation in cells as in (**A**). **D** Time-course evaluation of apoptosis by Incucyte caspase 3/7 apoptosis assay in cells as in (**A**). **E** Ratio of ATP/AMP peak intensities in cells as in (**A**). **F** BJ fibroblasts expressing MYC-ER^T2^ (MYC) or control (VEC) after 72 h of siRNA treatment and 24 h of asparagine supplementation. Apoptosis was assessed by annexin V/PI staining. An ordinary one-way ANOVA was used to determine statistical significance (*n* = 3 biological replicates). For all statistical test, *P* ≤ 0.05 was considered significant, and error bars show the standard error of the mean.

*MYC*-expressing glioma cells have previously been shown to undergo apoptosis upon glutamine withdrawal that can be rescued by asparagine supplementation. We compared the rescue from apoptosis afforded by glutamate and asparagine and observed that glutamate was far more effective in inhibiting apoptosis in 4-OHT-treated BJ fibroblasts expressing MYC-ER^T2^ (Fig. 6A). The lesser efficiency of asparagine in the apoptotic rescue was not due to a lack of uptake, since levels of asparagine present in the cells were over three orders of magnitude higher than those normally present (Fig. 6B). This indicates that availability of asparagine is not a limiting factor in the apoptosis triggered by glutamine withdrawal and that asparagine and glutamate must rescue through distinct metabolic mechanisms. Glutamate was able to partially rescue levels of TCA metabolites, whereas asparagine was unable to do so (Fig. 6C). Furthermore, the kinetics of apoptosis between glutamate and asparagine were distinct, with glutamate rescuing from the outset, whereas asparagine provided a more marginal reduction throughout the time course (Fig. 6D). Mass spectrometry analysis indicated that asparagine supplementation was able to improve the ATP/AMP ratio (Fig. 6E), indicating an impact on energetics without anaplerosis of the TCA cycle. Since exogenous asparagine gave rise to such high levels of intracellular asparagine, we questioned whether it might rescue apoptosis through product inhibition of asparagine synthetase (ASNS). Asparagine is produced through the action of asparagine synthetase (ASNS) in a reaction that requires aspartate, glutamine and hydrolysis of ATP, all of which are limiting under our apoptotic conditions. We utilised siRNA to silence ASNS and thereby determine whether ASNS activity was promoting apoptosis (through consumption of energetically limiting metabolites) or inhibiting apoptosis (due to increased availability of asparagine). Silencing of ASNS was confirmed by gene expression (Supplementary Fig. 6a), and led to an equivalent inhibition of apoptosis compared to the addition of exogenous asparagine (Fig. 6F), indicating that asparagine biosynthesis does not protect from the apoptosis triggered by glutamine withdrawal and that exogenous asparagine inhibits apoptosis through product inhibition of ASNS.

### Mitochondrial energy production correlates with protection from apoptosis

This dependence upon the TCA cycle, combined with the energetic shortfall described above, led us to focus on mitochondrial energy production in the sensitisation to apoptosis observed in MYC-overexpressing cells. Since inhibition of the TCA cycle affects several biosynthetic pathways in addition to energy production, we sought a distinct target in order to ascertain the role of mitochondrial energetics in the apoptosis driven by MYC activation and glutamine withdrawal.

**Fig. 7:**
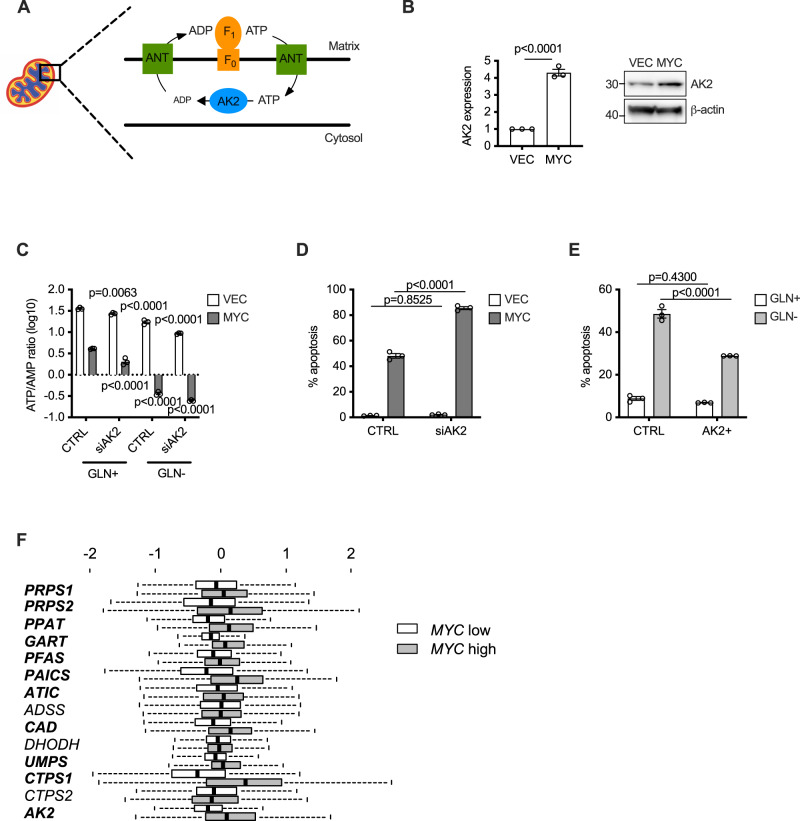
Facilitation of mitochondrial energy generation by AK2 limits MYC-induced apoptosis. **A** Schematic of the nucleotide environment in the mitochondrial intermembrane space where AK2 is located. **B** Induction of *AK2* expression by MYC shown by qRT-PCR (left panel) and western blot (right panel). Statistical analysis was carried out on *n* = 3 replicates using an unpaired two-tailed *t* test. **C** Ratio of peak intensities of ATP/AMP of MYC cells with knockdown of AK2 as measured by LC-MS. An ordinary one-way ANOVA was used to determine statistical significance (*n* = 3 biological replicates). **D**, **E** AnnexinV/PI assays in MRC-5 cells with (**D**) siRNA-mediated knockdown and (**E**) overexpression of AK2. An ordinary one-way ANOVA was used to determine statistical significance (*n* = 3 biological replicates). **F** Co-expression analysis (mean-centred log2 intensity) of *MYC* versus nucleotide biosynthesis and *AK2* genes derived from Affymetrix array data from 2999 breast cancers. The genes in bold are highly significant (*P* < 0.00001). Paired boxplots show the distributions of expression for each gene between two groups of microarray profiled breast tumours, MYC high and MYC low, based upon lower and upper tertiles of MYC expression (see 'Methods'). In each boxplot, the central point depicts the median value, the box bounds span Q1 to Q3, and left and right whiskers show Q1 − 1.5*IQR and Q3 + 1.5*IQR, respectively. For all statistical tests, *P* ≤ 0.05 was considered significant, and error bars show the standard error of the mean.

The classical model for the function of the adenylate kinases (AKs) is that they interconvert adenine nucleotides in a reversible reaction whereby two ADP molecules are converted into one ATP and one AMP molecule. Under conditions of low energy charge (e.g. through extensive hydrolysis of ATP to ADP or lack of ATP production), AKs can act as a source of ATP as well as generating AMP which activates AMP kinase, resulting in cellular energy conservation (reviewed in ref. 42). However, of the nine AKs, AK2 resides in a cellular compartment that experiences unique nucleotide metabolism—the mitochondrial intermembrane space. The adenine nucleotide translocase (ANT) on the inner membrane removes ADP into the mitochondrion and this transport is coupled with a return of ATP to the intermembrane space (Fig. 7A). This therefore favours the ‘reverse’ AK reaction, since a major substrate (ATP) is actively provided, whilst the product (ADP) is removed—a reaction that is further favoured upon conditions of AMP accumulation. By providing ADP for the mitochondrion to produce ATP, AK2 facilitates overall cellular ATP generation in keeping with the cellular function of the other members of this family. We observed that MYC activation led to an increase in AK2 mRNA and protein levels (Fig. 7B). Knockdown of AK2 reduced the ATP/AMP balance in all conditions tested (Fig. 7C), consistent with facilitation of mitochondrial ATP generation through the ‘reverse’ AK2 reaction described above. AK2 knockdown did not induce apoptosis in cells transduced with pM6P that were grown in a complete medium with 4-OHT but strongly enhanced apoptosis in cells transduced with pM6P-MYC-ER^T2^ in the absence of glutamine (Fig. 7D and Supplementary Fig. 6b, c), indicating that efficiency of nucleotide transfer between the cytosol and the mitochondrion limits the apoptotic response. Furthermore, overexpression of AK2 reduced the apoptosis caused by MYC activation and glutamine withdrawal (Fig. 7E and Supplementary Fig. 6d). Finally, we interrogated a large compendium of 2999 breast cancers to assess whether there was selective pressure to overexpress *AK2* in *MYC*-high tumours. Indeed, the level of *AK2* in *MYC*-high breast tumours was highly significantly elevated, as were other genes in the nucleotide biosynthesis pathways (Fig. 7F), indicating that the *AK2* gene exhibits positive selection in tumours expressing high levels of *MYC*. Thus, TCA cycle activity and mitochondrial energetics are critical determinants of the apoptosis triggered by glutamine withdrawal.

## Discussion

MYC requires its transactivation domain to induce apoptosis^2^, yet no specific transcriptional targets that mediate its apoptotic function have been found. An alternative possibility is that the global deregulation of transcription elicited by oncogenic MYC (accompanied by global activation of other energetically demanding processes such as translation) sensitises cells to apoptosis through a homeostatic mechanism, including or regardless of the primary transcriptional targets. Here we present data that support a homeostatic metabolic model for MYC-induced sensitisation to apoptosis. MYC induces metabolic demand for nucleotides that is strong enough to divert carbon away from glycolysis into a reversed PPP to support the production of ribose 5-phosphate and nucleotide biosynthesis. Our short-term stable isotope tracing studies with ^13^C_6_-glucose not only show enhanced flux through the oxidative arm of the PPP, but also demonstrate flux from glyceraldehyde 3-phosphate to ribose 5-phosphate through reversal of the non-oxidative arm of the PPP. This pathway consists of two bidirectional enzymes, transaldolase and transketolase, that catalyse reactions whose direction is determined by substrate & product concentrations. Thus, reversal of this pathway is a clear indicator of high demand for ribose 5-phosphate driven by MYC. It is also a striking example of how MYC can reprogramme cellular metabolism through a homeostatic mechanism separately from its direct transcriptional activation of metabolic target genes^43^.

The energy crisis that is driven by MYC activation and withdrawal of glutamine does not manifest as a major reduction in ATP levels, but rather as an accumulation of lower-energy nucleotides, nucleosides and bases. Within the nucleotide pools, this remodelling is most likely mediated by the adenylate kinases (with the exception of AK2) that catalyse the reversible conversion of 2 × ADP into ATP and AMP, allowing for the maintenance of ATP concentration whilst accumulating lower-energy species. Here we report that AK2 catalyses the opposite reaction, resulting in ADP production from AMP and ATP. This observation, whilst initially unexpected, is in fact predictable due to the localisation of AK2 to the intermembrane space. The adenine nucleotide translocase (ANT) on the inner mitochondrial membrane provides ATP to the intermembrane space whilst withdrawing ADP from this environment, promoting the production of ADP from ATP and AMP by AK2, particularly under conditions where cytosolic AMP is elevated. Through the generation of ADP, AK2 can therefore fuel mitochondrial ATP production and act to increase cellular energy charge. This non-canonical direction of AK2 catalysis is not only consistent with substrate/product availability but also with the physiological function of the gene family in the conservation of cellular energy charge.

The exact reasons for induction of nucleotide catabolism in our experiments, and indeed upon muscle activity^30,33^ and hypoxia^31^ remain obscure. We were unable to identify conditions under which inhibition of nucleotide catabolism was detrimental to cell survival (either through siRNA targeting of individual nucleotidases or usage of the XDH inhibitor allopurinol). This may, in large part, be due to redundancy in the catabolic pathways since there are eight currently identified nucleotidases in addition to the nine adenylate kinases that remodel adenine nucleotides in different cellular compartments. A plausible explanation for the catabolism and production of hypoxanthine and xanthine in such cases would be that accumulation of AMP leads to the promotion of nucleotidase activity purely based upon substrate concentration. Such a model would allow for catabolism of AMP and therefore continued activity of the AKs, enabling continued conversion of ADP to ATP in turn. Irrespective of potential benefit to cellular homeostasis, the products of nucleotide catabolism are diagnostic of the shortfall between energetic supply and demand in this context, as they are in others^31,33^.

Re-addition of glutamine rescued Myc-overexpressing cells from the apoptotic phenotype and enabled their proliferation, consistent with a homeostatic mechanism based on metabolic supply and demand rather than triggering of an irreversible commitment to apoptosis and cell cycle arrest. In contrast, the rescue from apoptosis afforded by asparagine is not accompanied by proliferation^34^ since there is a remaining requirement for glutamine to support multiple metabolic pathways. Our apoptotic and energetic data, supported by measurements of very high levels of asparagine, indicate that asparagine supplementation rescues from apoptosis through product inhibition of ASNS. Although ASNS activity consumes ATP, it seems unlikely that this could have a major impact on cellular energetics. We propose that a more likely explanation for this is that ASNS removes aspartate from the malate aspartate shuttle and that, in the absence of anaplerosis from glutamine, this has a detrimental impact on mitochondrial energetics.

Inhibition of glutaminase (specifically GLS1) was initially proposed as a promising strategy for targeting of MYC-driven tumours^44^ and the dependence of MYC expression upon glutamine availability in activated T cells also suggests that glutamine metabolism could be targeted in cancer^45^. Unfortunately, however, the therapeutic efficacy of glutaminase inhibition in Myc-driven tumours is rarely observed in vivo. Indeed, metabolism of glutamine via amidotransferases and altered utilisation of glucose-derived carbon have recently been identified as compensatory mechanisms in Myc-driven liver tumours^46^. This illustrates how metabolic redundancy can promote bypass of inhibition of single metabolic nodes and suggests that synthetic lethal approaches targeting multiple pathways are likely to be required. Since the reaction catalysed by OGDH has less redundancy than that catalysed by GLS, we targeted this enzyme in place of GLS inhibition. Genetic approaches cannot be used to recapitulate the metabolic impact of glutamine withdrawal because they lack the requisite rapidity of onset and so we turned to a small molecule inhibitor of OGDH, the lipoate analogue CPI-613. The mechanism of action of CPI-613 is likely to be complex, and it does not inhibit OGDH in all cases^38,40^, although we confirmed that it did so in our cells. Indeed, CPI-613 recapitulated the rapid apoptosis elicited by glutamine withdrawal in *MYC*-overexpressing cells in vitro and achieved tumour suppression in *MYC*-driven lymphoma in vivo. The development of more specific OGDH inhibitors may therefore be a desirable approach to therapeutic targeting of *MYC*-overexpressing tumours.

Our data also indicate that targeting AK2 could be an attractive option for cancer therapy since it should preferentially target cells that exhibit high demand for energy. Indeed, one can envisage a scenario where alternating regimes are applied, based upon inhibition of nucleotide biosynthesis (leading to selection for tumour cells that elevate biosynthetic capacity and therefore elevate metabolic demand) followed by inhibition of the TCA cycle and AK2 (leading to tumour suppression due to lack of energetic support for the excessive metabolic demand). Such ‘metronomic’ delivery of chemotherapy has been effectively demonstrated in a mouse model of melanoma^47^.

The robust impact of the BCL-2 family upon MYC-induced apoptosis indicates that common molecular mechanisms are most likely shared between the two. BCL-2 and BCL-xL can protect mitochondria from damage due to reactive oxygen species (ROS), mitochondrial membrane potential (MMP) decline and impaired nucleotide exchange and respiratory control^48–53^. BAX has been shown to require the mitochondrial ATPase for its apoptotic function^54^ and to localise to mitochondria in cardiomyocytes in response to ischaemia and AMPK activation^55^. The common denominator in most of these studies is the involvement of mitochondrial nucleotide metabolism in the regulation of apoptosis by the BCL-2 family, and our model of MYC-induced sensitisation to apoptosis through energetic demand continues that theme.

In summary, many oncogenes promote growth through activation of various signalling pathways or through transcriptional regulation of fundamental cellular programmes. The deregulation of these growth-promoting programmes inevitably has homeostatic consequences for the cell in terms of biosynthetic and energetic supply and demand. We propose that an increased understanding of these secondary homeostatic responses will open up new possibilities for therapeutic intervention.

## Methods

### Cells, treatments and siRNA

MRC-5 human fibroblasts (catalogue number 05072101 from Public Health England) were transduced with retroviral vector pM6P-blast (kind gift of F. Randow, MRC Laboratory of Molecular Biology, Cambridge UK) or pM6P-blast-MYC-ER^T2^ and selected with blasticidin (5 μg/ml). BCL-xL was expressed with pMSCV-based retrovirus. Cells were cultured in DMEM/10% FCS. Compounds for treatment were obtained from Sigma unless otherwise stated. 4-hydroxytamoxifen (200 nM) was used to activate the MYC-ER^T2^ and was present in all vector control samples as well. For siRNA experiments, DharmaFect 1 transfection reagent was used to deliver Dharmacon SMARTpool siRNA mix by reverse transfection in six-well plates at 30 nM final concentration. The day after transfection, assays were carried out as described below.

### AnnexinV/PI apoptosis assays

Cells were seeded in 6-well plates and on the following day, 4-OHT was added to all wells. After a further 24-h incubation, glutamine was withdrawn (where stated), and cells were incubated overnight. For determination of apoptosis, cells were trypsinized and collected into FACS tubes. Pellets were washed with PBS and resuspended in 100 μl annexin V buffer (10 mM HEPES pH 7.4, 140 mM NaCl, 2.5 mM CaCl_2_) and 5 μl annexin V-FITC (BD Bioscience). Immediately prior to analysis, propidium iodide was added. Data were acquired using a BD Accuri C6 flow cytometer and analysed using FlowJo v9.6.2, reporting single- and double-positive populations as apoptotic. The gating strategy is provided in Extended Data 1a.

### Incucyte apoptosis assays

Cells were seeded and treated as above, but at the point of amino acid withdrawal, Caspase 3/7 reagent (Essen Bioscience, UK) was added and apoptosis was measured using an Incucyte live cell imager (Essen Bioscience, UK). Results were obtained as percent apoptotic area based upon the percentage of cell area (determined by phase contrast microscopy) that stained positive for the caspase 3/7 reagent.

### EU transcription assay

A Click-iT^®^ RNA Alexa Fluor^®^ 488 HCS Assay (ThermoFisher) was used for the determination of transcription. Cells were plated in 96-well plates, treated as described, incubated with 5-ethynyl uridine (EU) for half an hour before fixation and permeabilization. Alexa Fluor^®^ azide was added for EU detection. Cells were then washed with Click-iT^®^ reaction rinse buffer and counterstained by DAPI. All the images and analysis were done with the ImageXpress Micro XLS Widefield High-Content Analysis System (Molecular Devices LLC, CA).

### Shotgun proteomic analysis of nascent translation

Following labelling of cells for 6 hours with ^13^C_6_,^15^N_2_-lysine, cell pellets were extracted with 8 M urea. DTT was added to 10 mM and samples were incubated at RT for an hour. Iodoacetamide was then added to 25 mM and incubated at RT for an hour and DTT was then added to final concentration of 25 mM. Samples were incubated overnight at 37*C with lysC (estimated ratio of lysC to the protein of 1:50 to 1:200) and peptides were acidified by addition of TFA to 0.5%. An estimated 10 μg of the resulting peptide solution was loaded onto an activated (20 μl methanol), equilibrated (100 μl 0.1% TFA) C18 StAGE tip and washed with 100 μl 0.1% TFA. The bound peptides were eluted into a Protein LoBind 1.5-ml tube (Eppendorf) with 20 μL 80% acetonitrile, 0.1% TFA and concentrated to less than 4 μl in a vacuum concentrator. The final volume was adjusted to 6 μl with 0.1% TFA. Online LC was performed using a Dionex RSLC Nano HPLC. Following the C18 clean-up, 5 μg peptides were injected onto a C18 packed emitter and eluted over a gradient of 2–80% ACN in 120 minutes, with 0.1% acetic acid throughout. Eluting peptides were ionised at +2 kV before data-dependent analysis on a Thermo Q-Exactive Plus. MS1 was acquired with *mz* range 300–1650 and resolution 70,000, and top 12 ions were selected for fragmentation with normalised collision energy of 26, and an exclusion window of 30 s. MS2 were collected with resolution 17,500. The AGC targets for MS1 and MS2 were 3e6 and 5e4, respectively, and all spectra were acquired with 1 microscan and without lockmass. Data were analysed using MaxQuant (v 1.5.8.3)^56^ in conjunction with UniProt FASTA database (UP000005640_9606.fasta (Human) 2017_05 release), with match between runs (MS/MS not required), LFQ with 1 peptide required, and statistical analyses performed in R^57,58^.

### Western blots

Cells were lysed in Cell Lysis Buffer (New England Biolabs) supplemented with Complete Protease Inhibitors (Roche) and subjected to SDS-PAGE, and transfer to Immobilon-P membrane (Merck-Millipore). After blocking in the blocking buffer (TBST + 5% non-fat milk), primary and HRP-conjugated secondary antibodies (Cell Signaling catalogues 7074S and 7076S) were applied in the blocking buffer and antigens were revealed with SuperSignal West Pico chemoluminescent reagent (ThermoFisher). Antibody to AK2 (HPA018479) was from Cambridge Biosciences. Antibody to c-MYC (clone Y69—ab32072) was from Abcam. Anti-phospho-AMPK^Thr172^ (Cell Signaling catalogue #2535), anti-AMPK (Cell Signaling catalogue #5831), anti-β-actin (13E5—Cell Signaling catalogue #4970S), and for Fig. 3E anti-c-MYC (clone 9E10—Novus Biologicals #NB600-302).

### RT-qPCR

RNA was extracted using an RNEasy Mini kit (Qiagen) and reverse transcribed using qScript cDNA Supermix (Quanta Biosciences) according to the manufacturer’s protocol. qPCR was performed on a StepOne RT-PCR system (ThermoFisher) using Sybr Select Master Mix (Life Technologies) and the following oligonucleotides: AK2 (tggtagtggagctcattgaga and catttctgcctgcctcaca), ASNS (aaaggtggcagatcatattg and atatgacttcatccagagcc).

### Extraction of metabolites

Metabolites were extracted from six-well plates by washing individual wells with ice-cold PBS and adding of cold extraction buffer (50% methanol, 30% acetonitrile, 20% water solution at −20 °C or lower). Extracts were clarified and stored at −80 °C until required.

### LC-MS

LC-MS was carried out using a 100 mm × 4.6 mm ZIC-pHILIC column (Merck-Millipore) using a Thermo Ultimate 3000 HPLC inline with a Q-Exactive mass spectrometer. A 32 min gradient was developed over the column from 10% buffer A (20 mM ammonium carbonate), 90% buffer B (acetonitrile) to 95% buffer A, 5% buffer B. 10 μl of metabolite extract was applied to the column equilibrated in 5% buffer A, 95% buffer B. Q-Exactive data were acquired in negative mode (or with polarity switching for the X13CMS analysis), and standard ESI source and spectrometer settings were applied (typical scan range 75-1050). Metabolites were identified based upon m/z values and retention time matching to standards. Quantitation of metabolites and their isotopologs was carried out using AssayR version 0.1.4^59^.

### Xenograft models

In all, 1 × 10^6^ Raji cells were injected subcutaneously into the right flank of 8- to 10-week-old male NOD-SCID IL2Rγ-null (NSG) mice. After 2 days, the animals were treated daily with intraperitoneal injections of either vehicle control or 20 mg/kg CPI-613. Tumour weight and engraftment were analysed when animals were euthanized because burden became large (approaching 1.2 cm in any dimension—no mice exceeded these limits) or when animals showed signs of poor health. All mice were maintained in a standard SPF facility (12 light/12 dark cycle, 19–23 °C with 40–60% humidity). These animal studies have been regulated under the Animals (Scientific Procedures) Act 1986 Amendment Regulations 2012 following ethical review by the University of Cambridge Animal Welfare and Ethical Review Body (AWERB-PPL number P846C00DB). HALO digital pathology software (Indica Labs) was used to analyse the percentage of tumour using a random forest tissue classifier in samples from haematoxylin counterstained formalin-fixed, paraffin-embedded (FFPE) sections. This analysis was carried out by the Histopath Facility at the Cancer Research UK Cambridge Institute.

### Tumour gene expression analysis

A compendium of 2999 breast tumours from 17 Affymetrix microarray studies was integrated as described previously^60^. *P* values are from *T* tests comparing means of lower and upper tertiles of *MYC* expression.

### Statistics and reproducibility

GraphPad Prism software (Version 9.3.1) was used for statistical analyses unless otherwise indicated in the figure legends. Statistical tests are indicated in the figure legends throughout. For all experiments, *P* values ≤ 0.05 were considered to be significant. In the case of X13CMS analysis, *P* values were calculated using the Student’s *T* test as part of the X13CMS workflow in R. Experiments were performed a minimum of three times, with the exception of the X13CMS and xenograft analyses that were carried out once.

### Reporting summary

Further information on research design is available in the Nature Research Reporting Summary linked to this article.

## Supplementary information

Supplementary Information

Reporting Summary

## Data Availability

The mass spectrometry proteomics data have been deposited to the ProteomeXchange Consortium via the PRIDE partner repository with the dataset identifier PXD034517. Raw metabolomics data are not included with this paper because they are used as an ongoing source of data. However, they can be obtained upon request from the corresponding author. Source data are provided with this paper.

## Source data

Source Data
