# Supplementary material for: MYC sensitises cells to apoptosis by driving energetic demand
Source: Nat Commun. 2022 Aug 9;13:4674. doi: 10.1038/s41467-022-32368-z (PMC9363429; doi:10.1038/s41467-022-32368-z)

Supplementary Figure 1. Gating strategy and confirmation of apoptosis in MRC-5 and Eμ-Myc lymphoma cells

Referring to Figure 1. **(a)** Gating strategy for all annexin V/PI apoptosis assays measured by flow cytometry. **(b)** MRC-5 cells overexpressing MYC-ER^{T2} (Myc) or MYC-ER^{T2} and Bcl-xL (Myc/Bcl-xL) were stimulated for 24 h with 200nM 4-OHT and glutamine was then withdrawn for 16 h. Apoptosis was assessed by annexinV/PI staining. **(c)** Annexin V/PI was used to quantify apoptosis in Eμ-Myc lymphoma cells in complete medium (+Gln) or following glutamine withdrawal for 16 h (-Gln). **(d)** Low magnification phase contrast images of VEC and MYC cultures after 24 h of glutamine withdrawal, after 24 h withdrawal and 24 h addback of glutamine and after 24 h withdrawal and 96 h addback of glutamine.

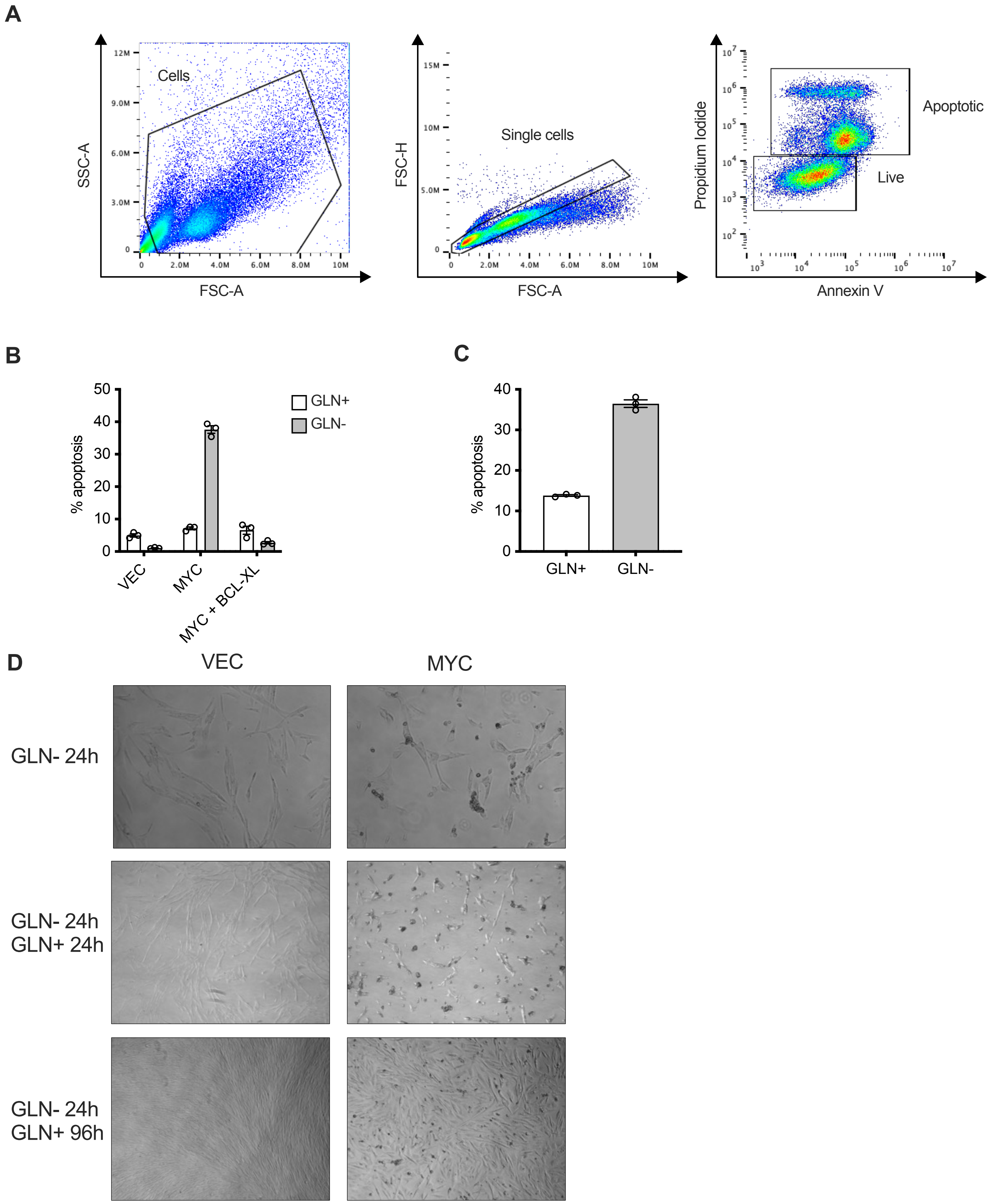

Supplementary Figure 2. Untargeted stable isotope tracing in MYC-expressing cells

Referring to Figure 2. Untargeted X13CMS analysis compared stable isotope incorporation in MRC-5 primary human fibroblasts with active MYC to empty vector control. Figure summarizes X13CMS analysis 'hits', which were defined as metabolites with significantly increased percent label incorporation ($p \leq 0.05$, T-test) from a 6 hr $^{13}C_6$-glucose pulse (blue boxes) or a 6 hr ^{15}N-amide-glutamine pulse (red boxes). Further targeted analyses were carried out on pathways containing hits to identify metabolites that may have been missed from the initial analysis (highlighted in blue for $^{13}C_6$-glucose and orange for ^{15}N-amide-glutamine).

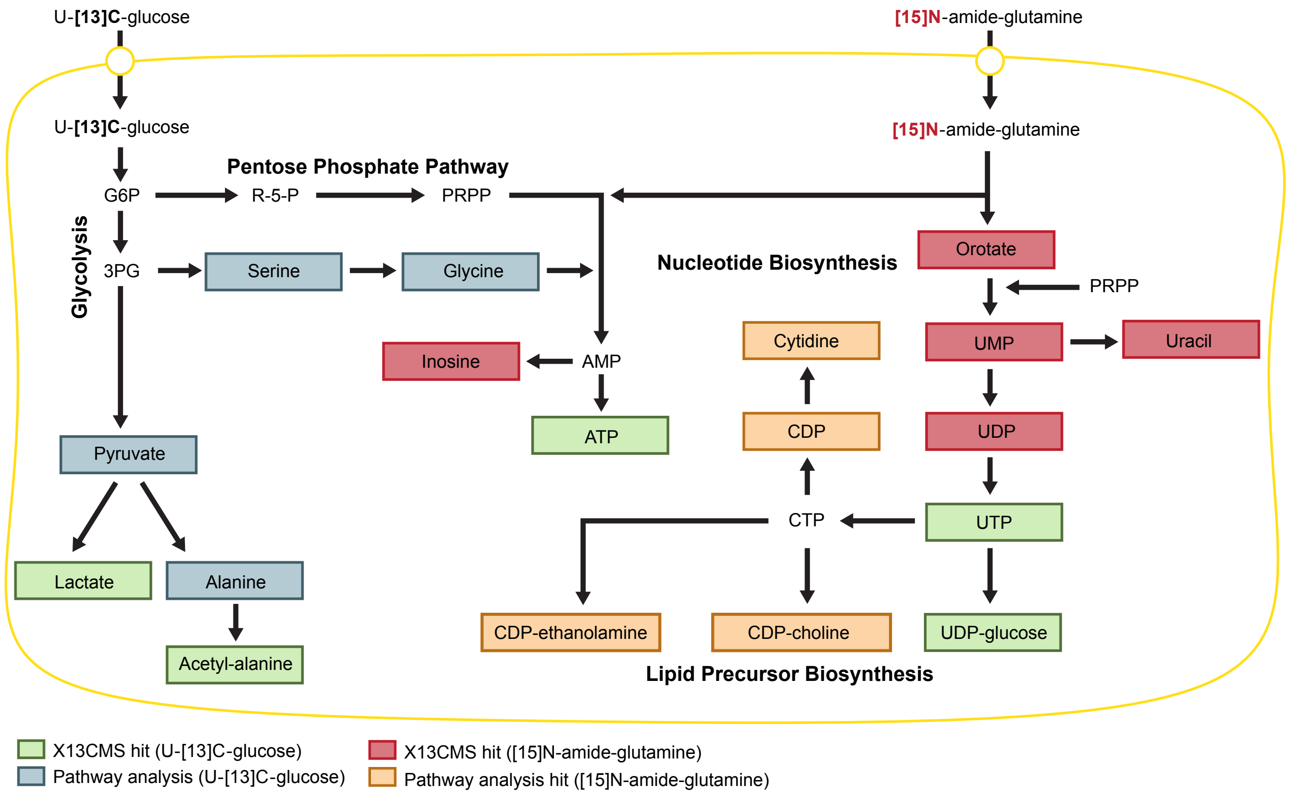

Supplementary Table 1. Untargeted X13CMS analysis of U-^{13}C-glucose incorporation

Referring to Figure 2. Untargeted X13CMS analysis compared stable isotope incorporation in MRC-5 primary human fibroblasts with active MYC to empty vector control. Table shows X13CMS analysis 'hits', which were defined as metabolites with significantly increased percent label incorporation (p≤0.05, T-test) from a 6 hr $^{13}C_6$-glucose pulse.

**X13CMS hits**			
Increased in Myc			
Metabolites	Predominant isotopologue	Ratio	p-value
Lactic acid	M+3	1.5	0.002
Acetyl-alanine	M+5	2.0	0.000
Glutamic acid	M+2	1.5	0.001
Acetyl-glutamic acid	M+2	11.7	0.000
Acety-methionine	M+2	1.2	0.000
Uridine triphosphate	M+5	1.2	0.001
Adenosine triphosphate	M+6	1.2	0.000
Uridine disphosphate-glucose	M+11	1.1	0.000
Decreased in Myc			
Acetyl-aspartyl-glutamic acid	M+11	10.57	0.000
**Normalised X13CMS hits**			
Increased in Myc			
Metabolites	Predominant isotopologue	Ratio	p-value
Lactic acid	M+3	1.4	0.010
Acetyl-alanine	M+5	1.9	0.000
Glutamic acid	M+2	1.5	0.004
Acetyl-glutamic acid	M+2	3.0	0.000
Acety-methionine	M+2	1.2	0.000
Uridine triphosphate	M+5	1.1	0.000
Adenosine triphosphate	M+6	1.2	0.000
Uridine disphosphate-glucose	M+11	1.1	0.000
Decreased in Myc			
Acetyl-aspartyl-glutamic acid	M+11	5.62	0.000
**Normalised pathway analysis hits**			
Increased in Myc			
Metabolites	Predominant isotopologue	Ratio	p-value
Pyruvic acid	M+3	1.3	0.000
Alanine	M+3	1.5	0.000
Glycine	M+2	6.0	0.000
Serine	M+3	4.2	0.000

Supplementary Table 2. Untargeted X13CMS analysis of ^{15}N-glutamine incorporation

Referring to Figure 2. Untargeted X13CMS analysis compared stable isotope incorporation in MRC-5 primary human fibroblasts with active MYC to empty vector control. Table shows X13CMS analysis 'hits', which were defined as metabolites with significantly increased percent label incorporation ($p \leq 0.05$, T-test) from a 6 hr ^{15}N-amide-glutamine pulse.

**X13CMS hits**			
Metabolites	Predominant isotopologue	Ratio	p-value
Uracil	M+1	Inf. (0 in control)	
Orotic acid	M+1	Inf. (0 in control)	
Cys-Gly	M+1	Inf. (0 in control)	
Inosine	M+1	Inf. (0 in control)	
Uridine monophosphate	M+1	Inf. (0 in control)	
Uridine diphosphate	M+1	Inf. (0 in control)	
Uridine diphosphate-acetyl-glucosamine	M+1	1.2	0.033
**Normalised X13CMS hits**			
Metabolites	Predominant isotopologue	Ratio	p-value
Uracil	M+1	29.5	0.000
Orotic acid	M+1	2.1	0.005
Cys-Gly	M+1	Inf. (0 in control)	
Inosine	M+1	Inf. (0 in control)	
Uridine monophosphate	M+1	Inf. (0 in control)	
Uridine diphosphate	M+1	3.3	0.000
Uridine diphosphate-acetyl-glucosamine	M+1	1.2	0.000
**Normalised pathway analysis hits**			
Metabolites	Predominant isotopologue	Ratio	p-value
Uridine triphosphate	M+1	1.5	0.000
Cytidine diphosphate	M+1	Inf. (0 in control)	
Cytidine	M+1	10.9	0.000
Cytidine diphosphate-choline	M+1	Inf. (0 in control)	
Uridine disphosphate-glucose	M+1	2.3	0.000
Cytidine diphosphate-ethanolamine	M+1	10.2	0.000

Supplementary Figure 3. The energetic effects of PPP inhibition occur prior to onset of apoptosis

Referring to Figure 3. **(a)** Ratio of peak intensities of ATP/AMP of VEC and MYC cells upon glutamine withdrawal for one hour. **(b)** Ratio of peak intensities of ATP/AMP of MYC cells upon inhibition of the PPP with 250 μM 6-AN for 2 hours with glutamine withdrawal for the second hour.

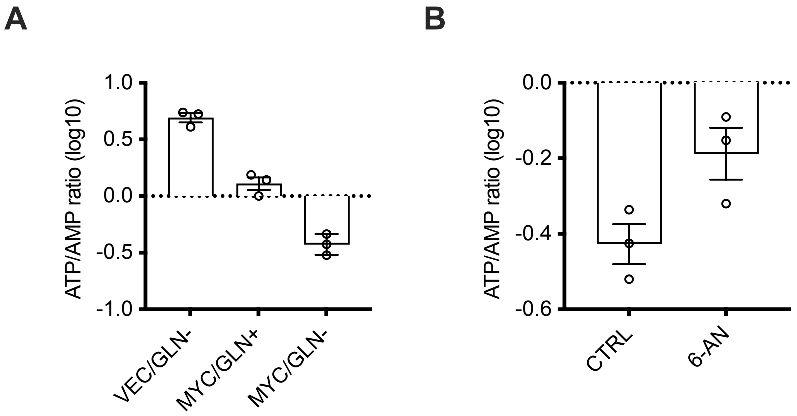

Supplementary Figure 4. MYC does not drive reductive carboxylation in MRC-5 fibroblasts

Referring to Figure 5. Isotopologue analysis of citrate following a $^{13}C_5$-glutamine pulse shows minimal reductive carboxylation compared to oxidative TCA cycling in MRC-5 VEC versus MYC cells.

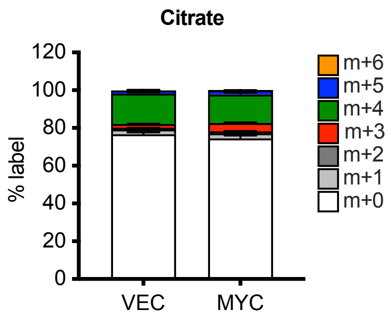

Supplementary Figure 5. Kinetics and succinate rescue of apoptosis triggered by CPI-613

Referring to Figure 5. **(a)** Kinetics of Incucyte caspase 3/7 analysis of MRC-5 cells triggered to undergo apoptosis by CPI-613. **(b)** Rescue of the apoptosis triggered by CPI-613 by addition of 4 mM dimethylsuccinate.

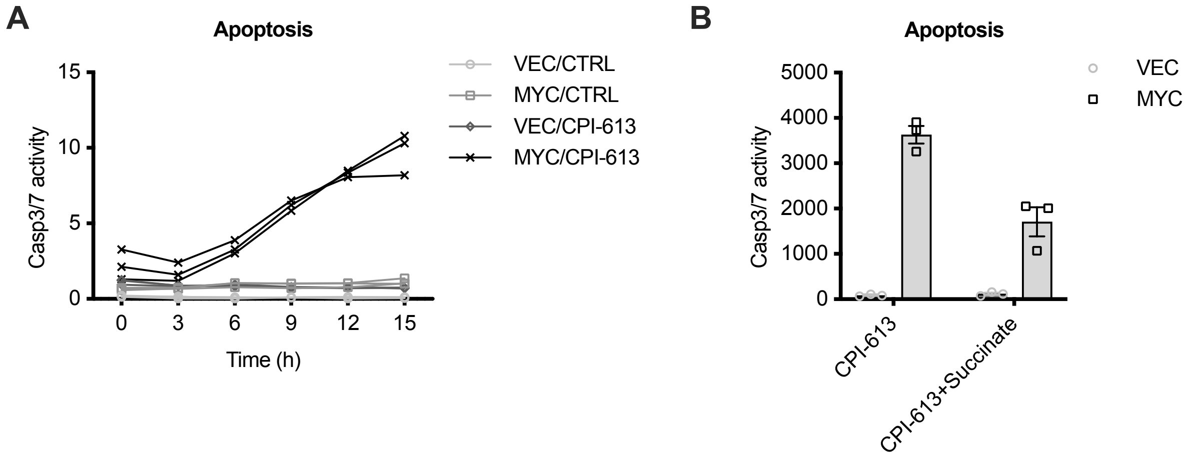

Supplementary Figure 6. Genetic manipulation of AK2 and ASNS

Referring to Figure 6. **(a)** qRT-PCR analysis of expression of *ASNS* (normalised to *GAPDH* mRNA) upon ASNS knockdown in MYC MRC-5 cells. Expression relative to VEC siNT is shown. **(b)** qRT-PCR analysis of expression of *AK2* (normalised to β-actin mRNA) upon AK2 knockdown (siAK2) or non-targeting control (siNT) in MRC-5 cells expressing MYC-ER^{T2}. Expression relative to siNT is shown. **(c)** Incucyte caspase 3/7 apoptosis assay in MYC cells with control siRNA (siNT) or siRNA to AK2 (siAK2) in medium containing glutamine or after 8 h of glutamine withdrawal. **(d)** qRT-PCR analysis of expression of *AK2* (normalised to *GAPDH* mRNA) upon overexpression of AK2 cDNA.

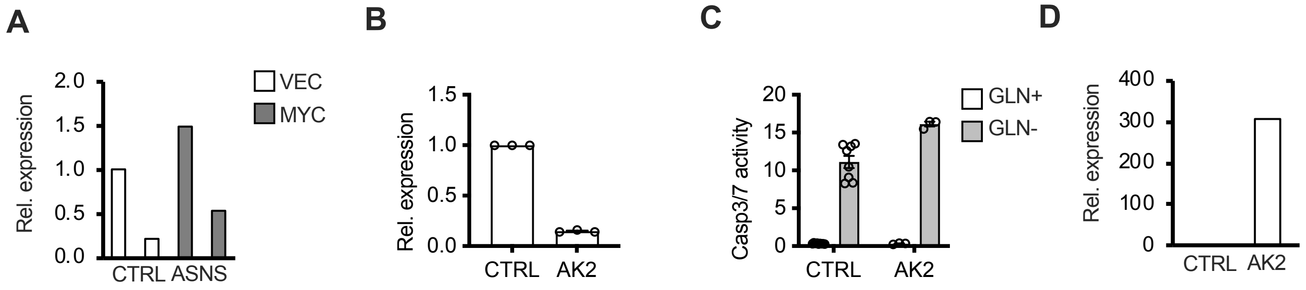

Supplement: Supplementary file 1 — Supplementary Information [file 41467_2022_32368_MOESM1_ESM.pdf]
