# Supplementary material for: MYC sensitises cells to apoptosis by driving energetic demand
Source: Nat Commun. 2022 Aug 9;13:4674. doi: 10.1038/s41467-022-32368-z (PMC9363429; doi:10.1038/s41467-022-32368-z)
Supplement: Supplementary file 2 — Reporting Summary [file 41467_2022_32368_MOESM2_ESM.pdf]

nature portfolio

Corresponding author(s): Andy Finch

Last updated by author(s): Jun 24, 2022

Reporting Summary

Nature Portfolio wishes to improve the reproducibility of the work that we publish. This form provides structure for consistency and transparency in reporting. For further information on Nature Portfolio policies, see our Editorial Policies and the Editorial Policy Checklist.

Statistics

For all statistical analyses, confirm that the following items are present in the figure legend, table legend, main text, or Methods section.

n/a	Confirmed	
☐	☒	The exact sample size (n) for each experimental group/condition, given as a discrete number and unit of measurement
☐	☒	A statement on whether measurements were taken from distinct samples or whether the same sample was measured repeatedly
☐	☒	The statistical test(s) used AND whether they are one- or two-sided<br>*Only common tests should be described solely by name; describe more complex techniques in the Methods section.*
☒	☐	A description of all covariates tested
☒	☐	A description of any assumptions or corrections, such as tests of normality and adjustment for multiple comparisons
☐	☒	A full description of the statistical parameters including central tendency (e.g. means) or other basic estimates (e.g. regression coefficient) AND variation (e.g. standard deviation) or associated estimates of uncertainty (e.g. confidence intervals)
☐	☒	For null hypothesis testing, the test statistic (e.g. F, t, r) with confidence intervals, effect sizes, degrees of freedom and P value noted<br>*Give P values as exact values whenever suitable.*
☒	☐	For Bayesian analysis, information on the choice of priors and Markov chain Monte Carlo settings
☒	☐	For hierarchical and complex designs, identification of the appropriate level for tests and full reporting of outcomes
☒	☐	Estimates of effect sizes (e.g. Cohen's d, Pearson's r), indicating how they were calculated

Our web collection on statistics for biologists contains articles on many of the points above.

Software and code

Policy information about availability of computer code

Data collection	Flow cytometry data were collected with BD Accuri C6 software (version unknown)<br>LC-MS data were acquired with XCalibur v2.2
Data analysis	Affymetrix array analysis was carried out as previously described (citation given in Methods).<br>Proteomic analysis was carried out in MaxQuant (v 1.5.8.3) in conjunction with UniProt FASTA database (UP000005640_9606.fasta (Human) 2017_05 release).<br>Metabolite analyses were carried out with AssayR version 0.1.4 (citation provided in Methods).

For manuscripts utilizing custom algorithms or software that are central to the research but not yet described in published literature, software must be made available to editors and reviewers. We strongly encourage code deposition in a community repository (e.g. GitHub). See the Nature Portfolio guidelines for submitting code & software for further information.

Data

Policy information about availability of data

All manuscripts must include a data availability statement. This statement should provide the following information, where applicable:

- Accession codes, unique identifiers, or web links for publicly available datasets
- A description of any restrictions on data availability
- For clinical datasets or third party data, please ensure that the statement adheres to our policy

Source data are provided with this paper. The mass spectrometry proteomics data have been deposited to the ProteomeXchange Consortium via the PRIDE partner repository with the dataset identifier PXD034517. Raw metabolomics data will be shared upon reasonable request.

Field-specific reporting

Please select the one below that is the best fit for your research. If you are not sure, read the appropriate sections before making your selection.

☒ Life sciences ☐ Behavioural & social sciences ☐ Ecological, evolutionary & environmental sciences

For a reference copy of the document with all sections, see nature.com/documents/nr-reporting-summary-flat.pdf

Life sciences study design

All studies must disclose on these points even when the disclosure is negative.

Sample size	Sample sizes are determined from experience. For metabolic analyses in vitro, typically 3 samples per condition are analysed.<br>For the in vivo experiments, cohorts of 6-7 were used (stated in the figure legend) based upon previous experiments.
Data exclusions	No data were excluded from these analyses
Replication	Experiments were replicated at least three times (much more in many cases) with good reproducibility, with the exception of the X13CMS analysis (extended data 2-4) and the xenograft experiments that were carried out once.
Randomization	Samples were distributed strategically (replicates were separated into distinct batches, inverted and shifted sample order between batches) at the point of mass spectrometric analysis to remove potential batch or temporal effects.
Blinding	No blinding was used for mass spectrometric analysis because of the need for careful sample ordering to remove batch and temporal effects (see above in 'Randomization'.<br>Mouse experiments were blinded until completion of data analysis.

Reporting for specific materials, systems and methods

We require information from authors about some types of materials, experimental systems and methods used in many studies. Here, indicate whether each material, system or method listed is relevant to your study. If you are not sure if a list item applies to your research, read the appropriate section before selecting a response.

Materials & experimental systems

n/a	Involved in the study	
☐	☒	Antibodies
☐	☒	Eukaryotic cell lines
☒	☐	Palaeontology and archaeology
☐	☒	Animals and other organisms
☒	☐	Human research participants
☒	☐	Clinical data
☒	☐	Dual use research of concern

Methods

n/a	Involved in the study	
☒	☐	ChIP-seq
☐	☒	Flow cytometry
☒	☐	MRI-based neuroimaging

Antibodies

Antibodies used	Antibody to AK2 (HPA018479) was from Cambridge Biosciences. Antibody to beta-actin (60008) was from Proteintech Group Ltd. Antibody to c-MYC (clone Y69 – ab32072) was from Abcam. Anti-phospho-AMPKThr172 (Cell Signaling catalogue #2535), anti-AMPK (Cell Signaling catalogue #5831), anti-β-Actin (13E5 - Cell Signaling catalogue #4970S), and for Fig 3b anti-c-MYC (clone 9E10 - Novus Biologicals #NB600-302).<br>HRP-conjugated secondary antibodies were Cell Signaling catalogues 7074S and 7076S.<br>This information is provided in Methods.
Validation	Myc antibody (Y69 - ab32072 from Abcam) is validated in the manuscript by RNAi and extensively validated by Abcam, including KO and blocking with a Myc peptide.<br>Anti-phospho-AMPKThr172 (Cell Signaling catalogue #2535) is validated by the manufacturer using oligomycin in C2C12 cells.<br>Anti-AMPK (Cell Signaling catalogue #5831) has been cited in hundreds of manuscripts.<br>Anti-c-MYC (clone 9E10 - Novus Biologicals #NB600-302) is validated in the manuscript by overexpression and has been extensively validated by the field.<br>AK2 antibody is validated by overexpression (in the manuscript) and knockdown (separately).<br>Anti-β-Actin (13E5 - Cell Signaling catalogue #4970S) is validated by the manufacturer through blotting for recombinant actin forms - it is specific for β-Actin.

Eukaryotic cell lines

Policy information about cell lines

Cell line source(s)	MRC5 primary human fibroblasts were obtained from ECACC via Public Health England.
Authentication	MRC5 primary human fibroblasts are authenticated by ECACC.
Mycoplasma contamination	Cell lines were regularly tested for Mycoplasma contamination and no incidents arose.
Commonly misidentified lines (See ICLAC register)	No commonly misidentified cell lines were used in this study.

Animals and other organisms

Policy information about studies involving animals; ARRIVE guidelines recommended for reporting animal research

Laboratory animals	The following information is provided in the Methods section:<br>8- to 10-week-old male NOD-SCID IL2Rγ-null (NSG) mice<br>Tumour weight and engraftment were analysed when animals were euthanized because burden became large (approaching 1.2 cm in any dimension – no mice exceeded these limits) or when animals showed signs of poor health. All mice were maintained in a standard SPF facility (12 light/12 dark cycle, 19–23°C with 40–60% humidity). These animal studies have been regulated under the Animals (Scientific Procedures) Act 1986 Amendment Regulations 2012 following ethical review by the University of Cambridge Animal Welfare and Ethical Review Body (AWERB-PPL number P846C00DB).
Wild animals	No wild animals were used in this study.
Field-collected samples	No field-collected samples were used in this study.
Ethics oversight	Experiments were regulated under the Animals (Scientific Procedures) Act 1986 Amendment Regulations 2012 following ethical review by the University of Cambridge Animal Welfare and Ethical Review Body (AWERB-PPL number P846C00DB).

Note that full information on the approval of the study protocol must also be provided in the manuscript.

Flow Cytometry

Plots

Confirm that:

☒ The axis labels state the marker and fluorochrome used (e.g. CD4-FITC).

☒ The axis scales are clearly visible. Include numbers along axes only for bottom left plot of group (a 'group' is an analysis of identical markers).

☒ All plots are contour plots with outliers or pseudocolor plots.

☒ A numerical value for number of cells or percentage (with statistics) is provided.

Methodology

Sample preparation	annexin V/PI samples from cultured cells were prepared as detailed in Methods.
Instrument	BD Accuri (described in Methods).
Software	FlowJo (described in Methods).
Cell population abundance	Flow sorting is not used in this manuscript.
Gating strategy	The gating strategy that was used is presented in extended data 1a.

☒ Tick this box to confirm that a figure exemplifying the gating strategy is provided in the Supplementary Information.
